# Toward a quantitative theoretical method for infrared and Raman spectroscopic studies on single-crystal electrode/liquid interfaces†

**DOI:** 10.1039/c9sc05429d

**Published:** 2019-12-10

**Authors:** Yuan Fang, Jin-Chao Dong, Song-Yuan Ding, Jun Cheng, Juan Miguel Feliu, Jian-Feng Li, Zhong-Qun Tian

**Affiliations:** State Key Laboratory of Physical Chemistry of Solid Surfaces (PCOSS), Collaborative Innovation Center of Chemistry for Energy Materials (iChEM), Department of Chemistry, College of Chemistry and Chemical Engineering, Xiamen University Xiamen 361005 China syding@xmu.edu.cn; Instituto Universitario de Electroquímica, Universidad de Alicante Carretera San Vicente del Raspeig s/n, E-03690 San Vicente del Raspeig Alicante Spain

## Abstract

*In situ* electrochemical infrared spectroscopy and Raman spectroscopy are powerful tools for probing potential-dependent adstructures at solid/liquid electrochemical interfaces. However, it is very difficult to quantitatively interpret the observed spectral features including potential-dependent vibrational frequency and spectral intensity, even from model systems such as single-crystal electrode/liquid interfaces. The clear understanding of electrochemical vibrational spectra has remained as a fundamental issue for four decades. Here, we have developed a method to combine computational vibrational spectroscopy tools with interfacial electrochemical models to accurately calculate the infrared and Raman spectra. We found that the solvation model and high precision level in the self-consistent-field convergence are critical elements to realize quantitative spectral predictions. This method's predictive power is verified by analysis of a classic spectroelectrochemical system, saturated CO molecules electro-adsorbed on a Pt(111) electrode. We expect that this method will pave the way to precisely reveal the physicochemical mechanism in some electrochemical processes such as electrocatalytic reactions.

## Abstract 2

An integrated approach for quantitatively predicting the electrochemical-infrared and electrochemical-Raman spectra and STM images of Pt(111)(2 × 2)-3CO adstructures has been developed.

## Graphical abstract

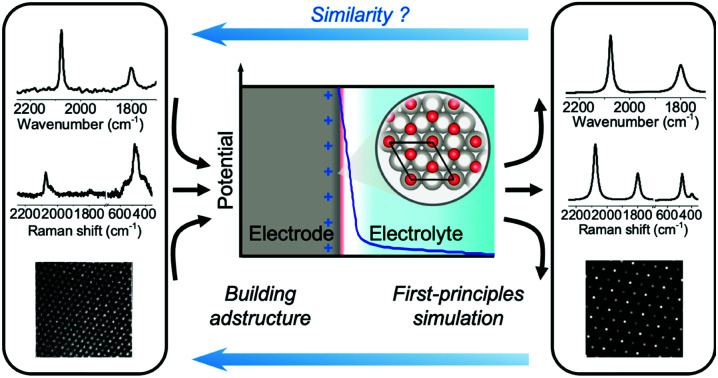

## Introduction

The determination of adstructures at electrochemical (EC) solid/liquid interfaces is a fundamental issue in fuel cells, metal/alloy plating and corrosion, *etc.*^1–3^ Vibrational spectroscopies can be utilized to provide fingerprint information about adstructures with high spectral resolution and have been developed for the characterization of EC interfacial adstructures by infrared (IR) spectroscopy since the mid-1960s^4^ and by Raman spectroscopy since the mid-1970s.^5^ Nevertheless, the vibrational frequencies and the intensities of EC-IR and EC-Raman spectra strongly depend on the applied potential, electrode materials, coverage of adsorbates and coadsorbed species, and thus are too complicated to be clearly interpreted in most of cases.^6,7^ For instance, with the Stark tuning slope (STS), the slope of the vibrational frequency as a function of the applied potential, it is difficult to precisely quantify potential-dependent behaviours of electroadsorption. Even for single-crystal electrodes with structurally well-defined surfaces, as perfect model systems for studying electroadsorption and electrocatalytic reactions,^8–14^ researchers still faced challenges in unambiguously assigning the vibrational modes in EC-IR and/or EC-Raman spectra. The above issue exposes the need for developing comprehensive and highly precise computational tools that facilitate the interpretation of EC-vibrational spectra.

First-principles computational methods based on cluster models with metal clusters to mimic the electrodes have been employed for calculating potential-dependent vibrational spectra of electroadsorbates.^15–18^ However, the cluster models in EC simulations are usually too simplified to exactly consider important effects on electrified single-crystal electrodes, such as the intermolecular interactions between electroadsorbates and periodic lattice structures. Therefore, slab models with periodic structures have been widely employed to study electroadsorption configurations and electrocatalytic mechanisms, especially for single-crystal or nanocrystal electrodes.^19–29^

At present, many studies have calculated the vibrational frequencies of adsorbates on slab models without the solvation model, which leads to the calculated STSs being much smaller than the experimental values.^30–33^ Moreover, studies have rarely calculated EC-IR or EC-Raman intensities,^34^ although crucial information, such as the orientation and coverage of adsorbates, adsorbate–adsorbate interactions, and charge-transfer interactions between adsorbates and substrates, could be extracted from the experimental analysis of the spectral intensities.^35,36^ Some efforts to understand the intensities were made based on the analysis of the symmetry of groups and the surface selection rule. These approaches can determine whether the vibrational modes of adstructures are either IR- or Raman-active, or both, but fail to predict potential-dependent relative and absolute intensities. In fact, accurately calculating EC-IR and EC-Raman intensities based on slab models by simultaneously considering the electrified surfaces and the surface solvation effect is a longstanding and difficult task.

**Scheme 1:**
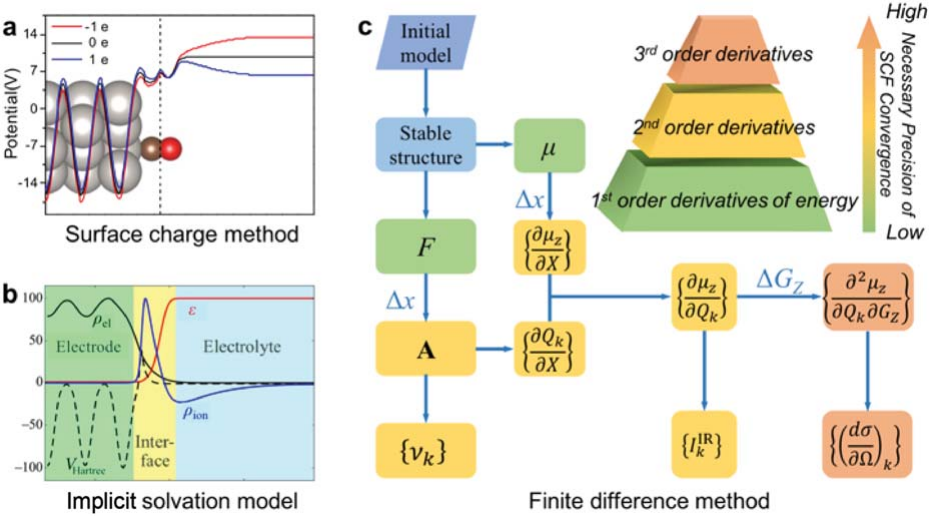
The computational method for EC-IR and EC-Raman spectra: (a) surface charged method, (b) implicit solvation model and (c) finite difference method. *μ*, *F*, *X*, *A*, *υ*_*k*_ and *G*_*Z*_ represent the surface dipole moment, atomic force, external atomic coordinates, dynamical matrix at the *Γ* point, vibrational frequency of the *k*^th^ normal-mode coordinate *Q*_*k*_, and external electric field along the *Z* axis, respectively. Δ*x* and Δ*G*_*Z*_ represent the step size of *X* and *G*_*Z*_ for the finite difference. Image (a) adapted from ref. 38 with permission, copyright (2011) Royal Society of Chemistry; image (b) adapted from ref. 29 with permission, copyright (2014) American Institute of Physics.

In this article, we report a new method for quantitatively predicting not only the vibrational frequencies but also the intensities of EC-IR and EC-Raman spectra from single-crystal electrodes. By coupling the surface charged method to mimic electrified surfaces (Scheme 1a),^22,37,38^ the implicit solvation model (Scheme 1b),^28,29^ and the finite difference method, the calculation of vibrational frequencies and intensities is successfully achieved (Scheme 1c; see also the ESI† for details).

## Results and discussion

### Simulation of EC-IR spectra of the Pt(111)(2 × 2)-3CO adstructure

**Fig. 1:**
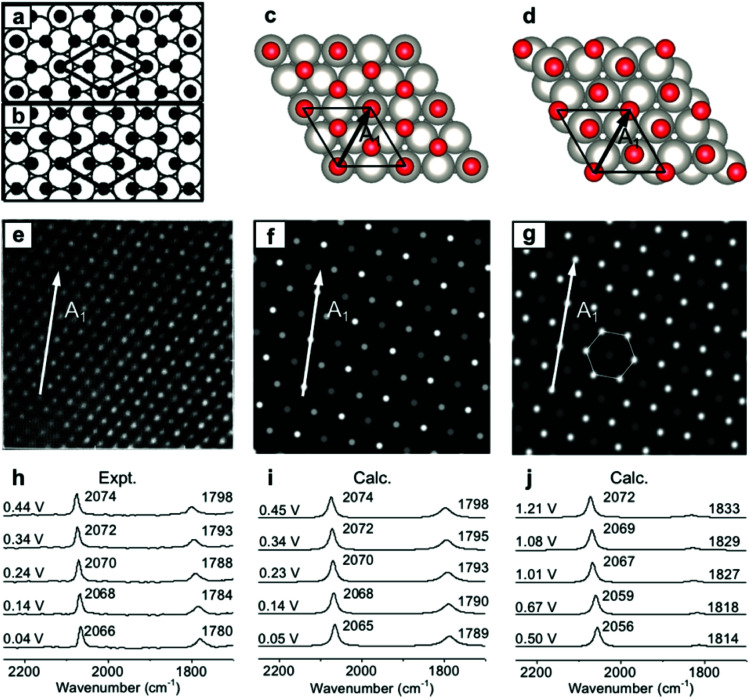
Pt(111)(2 × 2)-3CO adstructures at 0.04–0.44 V *vs.* SHE in a CO-saturated 0.1 M HClO_4_ aqueous solution: (a and b) the two possible adstructures (2 × 2)-3CO α_1_ and α_2_, respectively, proposed by Villegas *et al.*; (c and d) the respective optimized structures; (e) the measured EC-STM pattern;^44^ (f and g) the corresponding calculated patterns of the α_1_ and α_2_ structures; (h) the measured EC-IR spectra;^47^ and (i and j) the corresponding calculated spectra of the α_1_ and α_2_ adstructures. Images (a), (b) and (e) adapted from ref. 44 with permission, copyright (1994) American Institute of Physics; image (h) adapted from ref. 47 with permission, copyright (2000) American Chemical Society.

To validate the present computational method, we investigated the classic EC system of carbon monoxide (CO) adsorbed onto Pt(111) electrodes.^8,39–46^ Villegas and Weaver observed the Pt(111)(2 × 2)-3CO adstructure (Fig. 1e) with three CO molecules adsorbed onto a p(2 × 2) Pt(111) in a unit cell by performing EC-STM at potentials below 0.44 V *vs.* the standard hydrogen electrode (SHE) in a CO-saturated 0.1 M HClO_4_ aqueous solution.^44^ Two possible structures were proposed for the adstructure. One was Pt(111)(2 × 2)-3CO α_1_ with one CO molecule adsorbed at an atop site (CO_L_) and two CO molecules at hollow sites (CO_M_) in the unit cell (Fig. 1a). The other one was Pt(111)(2 × 2)-3CO α_2_ with a CO molecule adsorbed at a bridge site (CO_B_) and two CO molecules at near-top sites (Fig. 1b). Additionally, in experimental EC-IR spectra, the authors found a strong band from 2066 to 2074 cm^−1^ and a relatively weak band from 1780 to 1798 cm^−1^ (Fig. 1h). The two bands were assigned to the CO_L_ and CO_M_ stretching modes, respectively. Finally, they concluded that the most likely adstructure was the α_1_ rather than the α_2_ adstructure.^47,48^

In our calculated EC-IR spectra of the α_1_ structure (Fig. 1i), the bands centred at 2065–2074 cm^−1^ and 1789–1798 cm^−1^ are assigned to the stretching mode of one CO_L_ molecule and the in-phase stretching mode of two CO_M_ molecules, respectively. The localized or combinational CO stretching modes are shown in Fig. S1.† The calculated IR relative intensity *I*(CO_L_)/*I*(CO_M_) is *ca.* 1.2, which is close to the measured value of 1.5. Hence, the calculated vibrational frequencies and intensities of the α_1_ structure suitably agree with the values in the experimental EC-IR spectra. In addition, the calculated STM image in Fig. 1f contains one line of the brightest spots, one line of the second-brightest spots and one line of the darker spots along the *A*_1_ direction, which is consistent with the experimental image (Fig. 1e). The α_2_ adstructure was unstable in the potential range from 0.04 to 0.44 V *vs.* SHE. Moreover, the frequency of the CO_B_ stretching mode (*ca.* 1827 cm^−1^), the IR relative intensity *I*(CO_L_)/*I*(CO_B_) (4.1) and the STM pattern of the α_2_ adstructure (Fig. 1g and j) disagreed with the experimental observations. Therefore, this α_2_ structure was discarded.

### Importance of the solvation model for quantitatively predicting STSs of potential-dependent vibrational frequencies

Consideration of the solvation effect is a fundamental requirement for the reliable prediction of STSs. The STSs calculated from the CO_L_ and CO_M_ bands involving the implicit solvation model are 21 cm^−1^ V^−1^ and 24 cm^−1^ V^−1^, respectively. However, the calculated STSs of the two bands without the solvation model equal only *ca.* 1 cm^−1^ V^−1^ (Fig. S3a†), and compared with the measured slopes (22 cm^−1^ V^−1^ and 43 cm^−1^ V^−1^), these values are remarkably underestimated. We found that solvation could significantly increase the effective electrostatic field across the interfaces, further polarize the adsorbates, and finally result in much larger STSs than those in a vacuum. This role of solvation effect on STSs could also be elucidated by the monotonous increase in the absolute values of STSs as the increase in the relative permittivity *ε*_re_ of solvents as shown in Fig. S4b.† Furthermore, compared with the measured STS of the relatively weak band (43 cm^−1^ V^−1^), the calculated STS of the CO_M_ band (24 cm^−1^ V^−1^) is underestimated. This finding appears analogous to the “CO/Pt(111) puzzle”,^49,50^ which refers to the underestimated energy of the 2π* orbital from CO_M_ by DFT methods, and to the overestimation of the interaction between CO_M_ molecules and Pt substrates in a vacuum environment. Moreover, we found that the overestimated interaction was unaffected by the charged surface and the implicit solvation, which might in turn cause the underestimation of the STS of the CO_M_ band in the EC environment.

### Simulation of EC-Raman spectra of the Pt(111)(2 × 2)-3CO adstructure

**Fig. 2:**
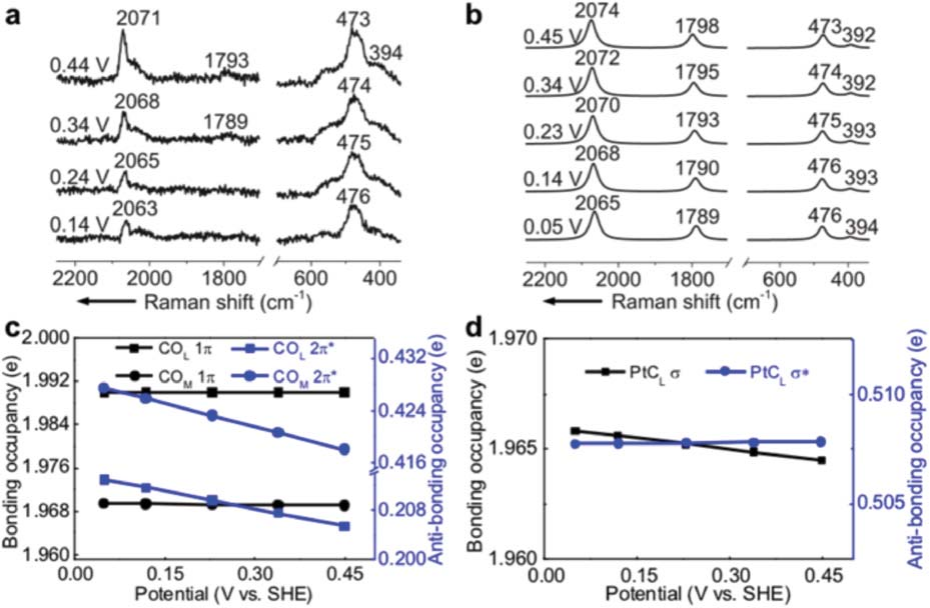
(a) Experimental EC-Raman spectra of a Pt(111) single-crystal electrode measured by SHINERS at 0.14–0.44 V *vs.* SHE in a CO-saturated 0.1 M HClO_4_ aqueous solution. (b) Calculated EC-Raman spectra of the (2 × 2)-3CO α_1_ adstructure. Potential-dependent NBO occupancies of the (c) 1π and 2π* orbitals of the CO_L_ and CO_M_ bonds and the (d) σ and σ* orbitals of the PtC_L_ bond. The slopes of the NBO occupancies are listed in Table S1.† The detailed NBO wavefunctions are listed in Fig. S6 and S7.†

EC-Raman spectroscopy is suitable for the characterization of vibrational modes occurring at low spectral frequencies. These vibrations directly reflect chemical complexation between adsorbed molecules and transition metal substrates. Therefore, we performed shell-isolated nanoparticle-enhanced Raman spectroscopy (SHINERS) on CO/Pt(111) EC interfaces in a CO-saturated 0.1 M HClO_4_ aqueous solution (Fig. 2a, experimental details in the Experimental section). The calculated vibrational frequencies and relative intensities of the Raman spectra of the α_1_ adstructure in Fig. 2b consistently agreed with the ones found by SHINERS. The Raman bands centred at *ca.* 2071, 1793, 473 and 394 cm^−1^ are assigned to the CO_L_, CO_M_, PtC_L_ and PtC_M_ stretching modes (Fig. S1†), respectively.

It is interesting to note that the STSs of the PtC_L_ and PtC_M_ bands are negative (−8 and −6 cm^−1^ V^−1^, respectively), while the CO_L_ and CO_M_ counterparts are positive, indicating that the CO bond strengthens and the PtC bond weakens as the potential increases. To understand the molecular mechanism behind this trend, we analysed the electronic structure of the α_1_ adstructure. The projected electronic densities of states (pDOSs) in Fig. S5a and b† show that the hybridized 5σ orbital of the CO molecule donates electrons to the Pt(111) surface to strengthen the PtC bonds, while the hybridized CO 2π* orbital accepts the back-donated electrons from the Pt d orbitals to weaken the CO bonds. Moreover, the natural bond orbitals (NBOs) in the periodic implementation were computed to quantitatively analyse the chemical-bonding response to the applied potential.^51^Fig. 2c and d show that the NBO occupancies of the CO 2π* and PtC_L_ σ orbitals shift negatively as the potential increases, which induces the strengthening of the CO_L_ and CO_M_ bonds and the weakening of the PtC_L_ bonds. In addition, the variations in the NBO occupancies of the 5σ, 5σ*, and 1π orbitals of CO and the PtC_L_ σ* orbital are insensitive to the applied potential (Table S1†).

### Importance of high-level precision in the SCF convergence for quantitatively predicting vibrational intensities

**Fig. 3:**
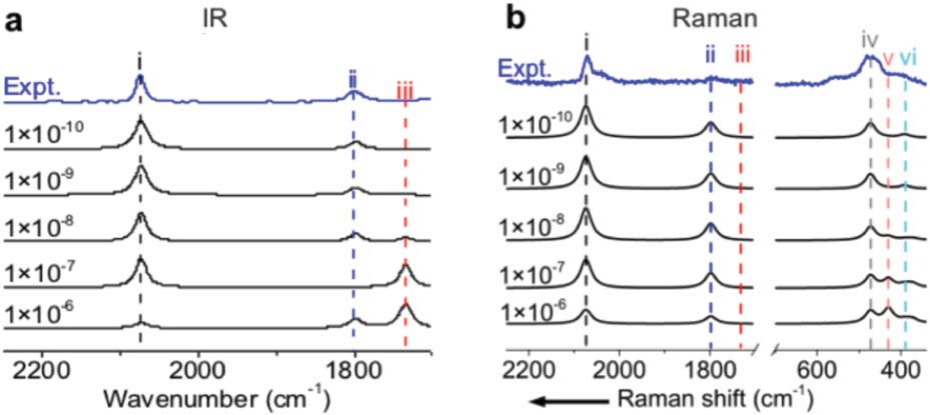
(a) Calculated EC-IR and (b) EC-Raman spectra of the (2 × 2)-3CO α_1_ adstructure at 0.45 V *vs.* SHE in a CO-saturated 0.1 M HClO_4_ aqueous solution under different SCF convergence criteria from 10^−6^ to 10^−10^ eV. The blue lines are the measured EC-IR in (a) and EC-Raman spectra in (b) at 0.44 V *vs.* SHE. The symbols (i), (ii), (iii), (iv), (v) and (vi) correspond to the CO_L_ stretching mode, symmetric CO_M_ stretching mode, antisymmetric CO_M_ stretching mode, PtC_L_ stretching mode, CO rotation mode and PtC_M_ stretching mode, respectively.

To precisely predict the vibrational intensities, the precision level of the convergence in the self-consistent-field (SCF) calculation (*ε*_SCF_) should be as high as 1 × 10^−9^ eV. For example, according to symmetry analysis, modes (ii) and (iii), assigned to the in-phase and out-of-phase combinational stretching modes of the two CO_M_ molecules, respectively, should originally be IR-active and IR-inactive. However, the two modes become pronouncedly IR-inactive and IR-active, respectively, if *ε*_SCF_ is 1 × 10^−6^ eV (Fig. 3a) which is the value by default in the calculation of vibrational frequencies. Similarly, several misleading relative intensities of Raman bands were predicted with larger *ε*_SCF_ values (Fig. 3b). This problem might be the reason for the scarce publications reporting the IR or Raman intensities at EC interfaces. In fact, all the spectra in Fig. 1 and 2 were calculated while including the implicit solvation model and an *ε*_SCF_ of 1 × 10^−9^ eV. With the as-developed method in hand, we can start predicting the EC-IR and EC-Raman spectra of some unknown adstructures at EC interfaces.

### Prediction of the EC-IR and EC-Raman spectra of metastable CO/Pt(111) adstructures

**Fig. 4:**
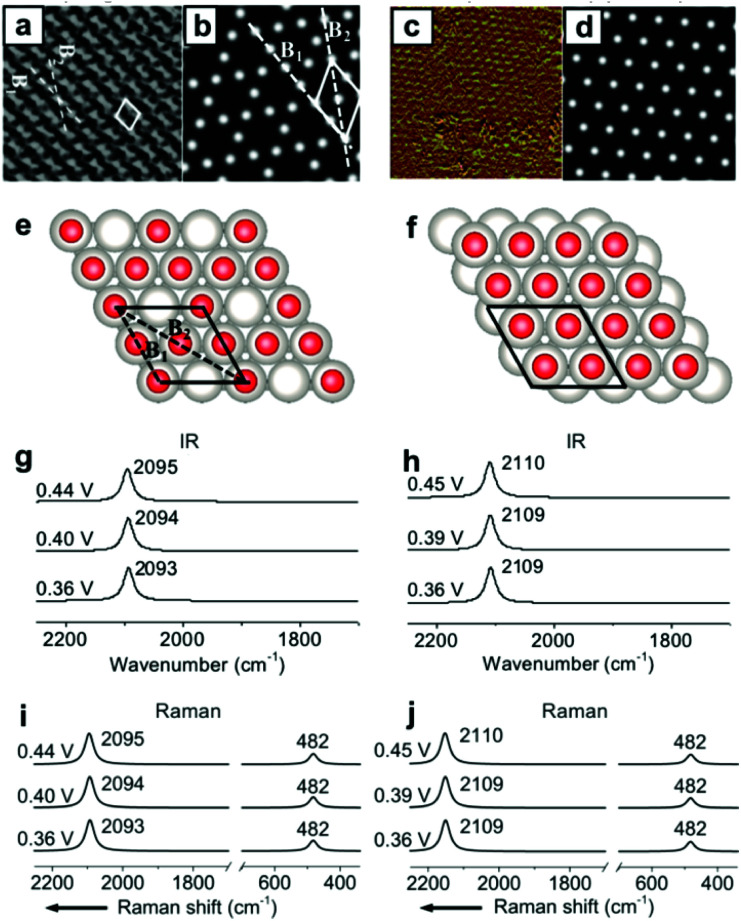
Two metastable adstructures at 0.36–0.45 V *vs.* SHE in a CO-saturated 0.1 M HClO_4_ aqueous solution: (a and c) the measured EC-STM images of the (2 × 2)-3CO β and (1 × 1)-CO adstructures, respectively;^45,46^ (b and d) the corresponding calculated EC-STM images; (e and f) the corresponding optimized adstructures; (g and h) the corresponding calculated EC-IR spectra; (i and j) the corresponding calculated EC-Raman spectra. Image (a) adapted from ref. 45 with permission, copyright (2006) Royal Society of Chemistry; image (c) adapted from ref. 46 with permission, copyright (2007) American Chemical Society.

Other than the well-known (2 × 2)-3CO α_1_ adstructure, Jung *et al.* proposed two unitarily atop-coordinated adstructures, (2 × 2)-3CO β and (1 × 1)-CO, at 0.36–0.45 V *vs.* SHE (Fig. 4a and c). However, neither the featured CO_L_ stretching bands (∼2094 cm^−1^ and ∼2110 cm^−1^) nor the PtC_L_ stretching band (482 cm^−1^) of the two adstructures in Fig. 4g–j can be observed in the measured IR spectra in Fig. 1h and the measured Raman spectra in Fig. 2a (∼2074 cm^−1^ and ∼474 cm^−1^).

Accordingly, the absolute values of the binding energies (|*E*_B_|) of the (2 × 2)-3CO β and the (1 × 1)-CO adstructures are approximately 0.39 and 0.59 eV smaller, respectively, than those of the (2 × 2)-3CO α_1_ adstructure (Fig. S8†), which indicates that the (2 × 2)-3CO β and (1 × 1)-CO adstructures are metastable and might occupy domains of the Pt(111) electrode down to the nanometer scale. Thus, the (2 × 2)-3CO α_1_ adstructure dominantly contribute to the EC-IR and EC-Raman intensities by wide-field spectroscopic measurement, while the two metastable adstructures might provide only a negligible contribution to the wide-field spectra and the vibrational bands assigned to the metastable adstructures cannot be observed. It would be desirable to characterize the metastable adstructures in the minute domains by employing tip-enhanced Raman spectroscopy (TERS) at solid/liquid EC interfaces with ultrahigh sensitivity and spatial resolution or by developing a method for nanoscale IR spectroscopy in liquid.

## Conclusions

A theoretical method combining the computational tools of vibrational spectroscopy and interfacial-EC models (surface charge method and implicit solvation model) was developed for quantitatively predicting EC-IR and EC-Raman spectra, not only the vibrational frequencies but also the spectral intensities. The validity of the combined method was well demonstrated by the study of the well-known Pt(111)(2 × 2)-3CO α_1_ adstructure. We demonstrated that the precision level of the convergence in the SCF calculation should be as high as 1 × 10^−9^ eV rather than the commonly used 1 × 10^−6^ eV for the quantitative prediction of the relative intensities of EC-IR and EC-Raman spectra. The as-developed method can be straightforwardly applied to reveal the electro-adstructures of general molecules on general single-crystal electrodes, such as bimetallic electrodes, semiconductor electrodes, and some emerging 2D material electrodes. In addition, the implicit solvation model may be further combined with explicit water molecules in the inner Helmholtz plane for more complicated reactant-water or oxyanion-water adstructures coadsorbed on electrified surfaces. This work may provide a new opportunity to deeply reveal reaction mechanisms mediated by coadsorbed water molecules and to study the potential-induced phase transition of a structured-water layer in future.

## Computational section

The metal surfaces were modelled as p(2 × 2) and p(√19 × √19) Pt(111) slabs with a lattice constant of 3.97 Å and a thickness of 7 layers in a periodic box of 108.75 Å (the middle three layers were frozen in its bulk position and detailed geometries are given in Appendix I of the ESI†). The first-principles computations were performed using the Vienna *ab initio* simulation package (VASP) with projector augmented wave (PAW) pseudopotentials.^52,53^ The alternative revision of the Perdew–Burke–Ernzerhof functional RPBE was employed to exactly calculate the chemisorption energies of CO molecules on the Pt(111) surfaces.^54^ We considered a 500 eV cut-off energy for reciprocal space mesh size and a 5 × 5 × 1 and 2 × 2 × 1 *Γ*-centred *K*-point mesh for p(2 × 2) and p(√19 × √19) unit cells, respectively. The SCF convergence criterion was set to 1 × 10^−9^ eV. The force of optimized geometries should be less than 0.01 eV Å^−1^ for converged vibrational frequencies of the systems (Table S2†).

The implicit solvation model implemented in VASPsol based on the linearized Poisson–Boltzmann equation (also named the linearized polarizable continuum model, LinearPCM) was employed to mimic the surface solvation effect (see the ESI† for details).^28^ The relative permittivity *ε*_re_ and Debye screening length *λ* were, respectively, set to 78 and 9.5 Å for ionic strength *I* = 0.1 M to describe the 0.1 M HClO_4_ aqueous solution in experiments. The calculated potential of zero charge (PZC) in this article under the adstructure √19 × √19 (a 0.68 monolayer) for the coverage (CO per surface Pt atoms), is 1.22 V *vs.* SHE and is close to the measured PZC, 1.1 ± 0.04 V.^55,56^ The precise prediction of PZC suggests that the implicit solvation model is reasonable for mimicking the CO/Pt(111) interface at a high CO coverage. In addition, more accurate implicit solvation models such as nonlinear electrochemical soft-sphere model should be employed for more accurate description of solid/liquid interfaces with low ionic strengths,^57,58^ because the LinearPCM model might fail to simulate the ionic response in low ionic strengths and the capacitance of solvent near PZC.

## Experimental section

We employed an Xplora Raman instrument to perform *in situ* spectroelectrochemical experiments. The excitation laser wavelength of the Xplora instrument was 638 nm (the laser power about 6 mW) and a 50× magnification long working distance (8 mm) objective was used. Before the Raman experiment, the Raman frequencies were calibrated using a Si wafer and the experimental Raman spectra of the experiment were collected during 60 s for a single-spectrum curve. A three-electrode spectroelectrochemical cell was employed for the electrochemical Raman experiment. A platinum wire was employed as a counter electrode and a saturated calomel electrode (SCE) was used as a reference electrode. A Pt(111) single-crystal electrode was used as a working electrode assembled with Au@SiO_2_ nanoparticles to enhance the Raman signal.^11,59^ The CO electro-adsorption experiment was performed in a CO-saturated 0.1 M HClO_4_ aqueous solution.

## Conflicts of interest

There are no conflicts to declare.

## Supplementary Material

SC-011-C9SC05429D-s001
