# Supplementary material for: Toward a quantitative theoretical method for infrared and Raman spectroscopic studies on single-crystal electrode/liquid interfaces
Source: Chem Sci. 2019 Dec 10;11(5):1425–30. doi: 10.1039/c9sc05429d (PMC8148070; doi:10.1039/c9sc05429d)
Supplement: SC-011-C9SC05429D-s001 [file SC-011-C9SC05429D-s001.pdf]

Electronic Supplementary Material (ESI) for Chemical Science.
This journal is © The Royal Society of Chemistry 2019

Supporting Information (SI)

Toward a quantitative theoretical method for infrared and Raman spectroscopic studies on single-crystal electrode/liquid interfaces

Yuan Fang,[a] Jin-Chao Dong,[a] Song-Yuan Ding,[a]* Jun Cheng,[a] Juan Miguel Feliu,[b] Jian-Feng Li,[a] and Zhong-Qun Tian[a]

[a] State Key Laboratory of Physical Chemistry of Solid Surfaces (PCOSS), Collaborative Innovation Centre of Chemistry for Energy Materials (iChEM), and Department of Chemistry, College of Chemistry and Chemical Engineering, Xiamen University, Xiamen 361005, China.
[b] Instituto Universitario de Electroquímica, Universidad de Alicante, Carretera San Vicente del Raspeig s/n, E-03690 San Vicente del Raspeig, Alicante, Spain.

*Corresponding author. E-mail: syding@xmu.edu.cn

Contents:

Computational details....................S2-S3

Surface charged method and free energy correction....................S3-S4

Implicit solvation model....................S4-S5

Calculation of the IR and Raman spectra....................S5-S6

Vibrational modes of (2×2)-3CO α_1 and α_2 adstructures....................S6-S7

Comparison between the calculated IR spectra in a vacuum and a solution....................S7

pDOS and NBO wavefunctions of (2×2)-3CO α_1 adstructure....................S8-S9

Potential-dependent binding energy of adstructures....................S10

Appendix I. Calculated geometries....................S11-S17

Computational details

The metal surfaces were modeled as symmetric p(2×2) and p(√19×√19) Pt(111) slabs with a lattice constant of 3.97 Å and a thickness of 7 layers in a periodic box of 108.75 Å (the middle three layers were frozen in its bulk position and detailed geometries were given in Appendix I). All computations were performed with VASP 5.4.1 package.[1] The electronic structure is described at the RPBE level,[2] with the cut-off energy of 500 eV for the plane-wave basis set. The electron-ion interactions were described by the projector augmented wave method (PAW).[3] The pseudo-wave functions, the smooth part of the charge density and the potential were represented on a 40×60×600 fast-Fourier-transform (FFT) mesh. The Brillouin zone was integrated by a 5×5×1 and 2×2×1 Γ-centered K-point mesh for p(2×2) and p(√19×√19) unit cells, respectively. All geometries were optimized to reach a gradient smaller than 0.01 eVÅ^{-1} with wave functions converged to 1×10^{-9} eV.

VASPsol implementation was employed to mimic surface solvation effect.[4, 5] The relative permittivity ε_r and Debye screening length λ were, respectively, set to 78 and 9.5 Å. The surface tension was set to zero (no cavitation energy).

Finite difference method was employed to calculate IR and Raman spectra. The differential step size of Cartesian coordinates Δx and electrostatic field strengths along the z axis ΔG_z were set to 0.01 Å and 0.1 V/Å, respectively. The calculated frequencies with respect to the measured ones were corrected by the linear inhomogeneous functions, $0.993\upsilon + 57$ cm^{-1} ($\upsilon \geq 1700$ cm^{-1}) and $0.851\upsilon + 65$ cm^{-1} ($\upsilon < 1700$ cm^{-1}). The calculated IR and Raman intensities were broadened by Lorentzian function with full

width at the half-maximum (FWHM) of 35 cm^{-1}.

Surface charged method and free energy correction

Electrified surface was simulated by the surface charged (SC) method, which was firstly reported by Filhol and Neurock.[6, 7] In this section, we briefly rewrote the computational method. The SC method makes systems charged by modifying n_0 valence electrons of systems to n_e electrons. For neutralizing the periodic cells, the homogeneous background charge was applied. The electrode potential of specific n_e referenced to standard hydrogen electrode V_{SHE}, is calculated by

$$V_{SHE} = \phi_{vac} - \phi_{SHE}, \quad (1)$$

where ϕ_{vac} is the absolute potential of specific n_e, which is equal to the workfunction of systems. ϕ_{SHE} is the absolute potential of the standard hydrogen electrode. IUPAC recommends to assign a value of $\phi_{SHE} = 4.44$ V.[8]

Due to the interaction between the charged slab and its compensating background charge, the total energies of adsorption systems E_{DFT} can be separated into three contributions in Eqn.2

$$E_{DFT}(n_e,n_{bg}) = E_{slab}(n_e) + E_{slab-bg}(n_e,n_{bg}) + E_{bg}(n_{bg}), \quad (2)$$

where E_{slab}, E_{bg} and $E_{bg\text{-}slab}$ are the energies of the slab without the background, that of the background without the slab, and that of the interaction between the slab and the background, respectively.[6, 9] The sum of latter two terms can be approximately given by

$$-\int_0^q \langle \overline{V_{tot}}(Q) \rangle dQ, \quad (3)$$

where $\langle \overline{V_{tot}}(Q) \rangle$ is the average potential in the unit cell. Thus, the corrected total

electron energy, E_{elec} could be defined as,

$$E_{elec} = E_{DFT} + \int_0^q \langle \overline{V_{tot}}(Q) \rangle dQ \tag{4}$$

,

The total free energy of the system E_{free}, including the contribution of excess electrons (q) at ϕ_{vac}, is given by

$$E_{free} = E_{DFT} + \int_0^q \langle \overline{V_{tot}}(Q) \rangle dQ - q\phi_{vac} \tag{5}$$

.

Based on the corrected energies at the same area of unit cell, the absolute value of potential-dependent binding energies $|E_B|$ of adsorption systems can be calculated by

$$|E_B| = \left| \frac{E_{free}^{system}(V) - E_{free}^{surface}(V) - nE_{molecule}}{n} \right| \tag{6}$$

,

where n is the number of molecules in the unit cell. Esystem free(V), Esurface free(V) and $E_{molecule}$ are the energies of adsorption system, surface and isolated molecules, respectively.

Implicit solvation model

Implicit solvation model for periodic DFT code was pioneered by Gygi *et al.*[10] VASPsol by Hennig *et al.*[4, 5] was employed to consider the implicit solvation effect. In brief, the solute was treated quantum-mechanically and the solvent was treated as a continuous medium based on Poisson-Boltzmann equation in VASPsol. The total energy, E_{DFT}, of the system consisting of the solute and solvent is expressed as

$$E_{DFT} \equiv E_{KS}[n(r)] + \Delta E[n(r), N(r), \phi(r)] \tag{7}$$

,

where E_{KS} term is the energy contribution of Kohn-Sham density functional for a solute system. The variables of n and N are, respectively, the electronic and nuclear charges of the solute system, and ϕ is the combined electrostatic potential. The ΔE term, is given

by

$$\Delta E = \frac{1}{2}\int \phi(r)\rho_{ion}(r)d^3r + \int |\nabla S|d^3r + kTS_{ion}$$
$$+ \left(\int \phi(r)\rho_{el}(r)d^3r - \int \epsilon(r)\frac{|\nabla\phi|^2}{8\pi}d^3r \right). \qquad (8)$$

ΔE includes the energy contributions of solvent, solute cavity, the entropy of mixing of the ions S_{ion}, and solute/solvent interaction terms. The parameter ρ_{el} is the sum of the solute electronic and nuclear charge densities, ρ_{ion} and ϵ are the ionic charge density and relative permittivity of the electrolyte. In the solute/solvent interface, these variables are scaled by a shape function

$$S(n(r)) = \frac{1}{2}erfc\left\{ \frac{log\left({}^{n}/_{n_c}\right)}{\sigma\sqrt{2}} \right\}. \qquad (9)$$

The parameter n_{c} is the electron density of the solute cavity, and σ is the diffuse width. Taking the variation of total energy, E_{DFT} with respect to ϕ yields the linearized Poisson-Boltzmann equation

$$\nabla\epsilon\nabla\phi = - \rho_{el} - \rho_{ion}. \qquad (10)$$

Calculation of the IR and Raman spectra

As shown in Scheme S1, finite difference method was employed throughout the whole calculation route. The vibrational frequencies $\{\nu_{\mathrm{k}}\}$ and normal coordinates of vibrational modes $\{\partial Q_{\mathrm{k}}/\partial X\}$ are derived from the eigenvalues and vectors of dynamical matrix **A**. The derivative of dipole moment μ, $\{\partial\mu/\partial X\}$ and **A** matrix can be approximately given by the difference of μ and force F along with atomic Cartesian coordinates X. Due to surface selection rule, we only use the partial dipole moment along with Z axis μ_{z} to calculate spectral intensity.

The IR spectra intensity IIR k for a specific mode Q_k can be calculated by

$$I_k^{IR} = \frac{N\pi d_k}{3c^2}\left|\frac{\partial \mu_z}{\partial Q_k}\right|^2 , \tag{11}$$

where N is Avogadro's number, d_k is the degeneracy of the kth mode and c is the speed of light.[11] The derivative of μ_z with respect to the kth mode $\partial\mu_z/\partial Q_k$, is given by

$$\frac{\partial \mu_z}{\partial Q_k} = \frac{\partial \mu_z}{\partial X}\frac{\partial X}{\partial Q_k}. \tag{12}$$

The Raman cross section for the Stokes component of the kth mode is given by

$$\left(\frac{d\sigma}{d\Omega}\right)_k = \frac{2\pi^2 h}{c\upsilon_k}\frac{(\upsilon_0 - \upsilon_k)^4}{1 - exp(-hc\upsilon_k/k_BT)}\frac{4\left(\frac{\partial^2 \mu_z}{\partial Q_k \partial G_z}\right)^2}{15} , \tag{13}$$

where h and k_B are Plank's and Boltzmann's constant and υ_0 is the frequency of incident light.[11] The $\{\partial^2\mu_z/\partial Q_k\partial G_z\}$ can be approximately calculated by the difference of $\{\partial\mu_z/\partial Q_k\}$ along with the Z axis electrostatic field strength G_z.

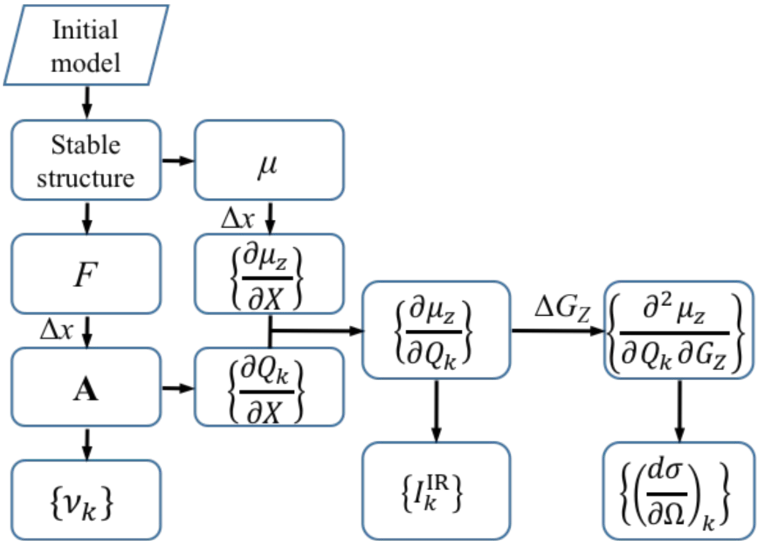

Scheme S1. Route diagram of IR and Raman spectra calculation.

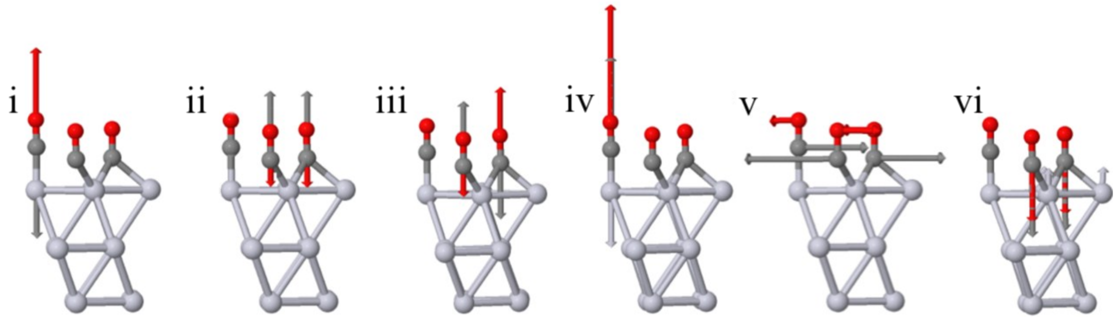

Fig. S1 Vibrational modes of the (2×2)-3CO α_1 adstructure: (i) denotes the CO_L stretching mode; (ii) and (iii) denote the symmetric and anti-symmetric CO_M stretching modes, respectively; (iv) denote the PtC_L stretching mode; (v) denotes CO rotation mode; (vi) denotes PtC_M stretching mode. The symmetric CO_M stretching mode is IR and Raman active, whereas the anti-symmetric CO_M stretching mode is IR and Raman inactive. The CO rotation mode is also IR and Raman inactive.

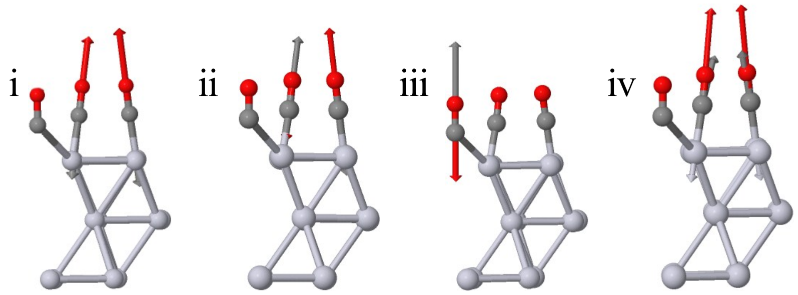

Fig. S2 Vibrational modes of the (2×2)-3CO α_2 adstructure: (i) and (ii) denote the symmetric and anti-symmetric CO_L stretching modes, respectively; (iii) denotes the CO_B stretching mode; (iv) denotes the PtC_L stretching mode. The symmetric CO_L stretching mode is IR active, whereas the anti-symmetric CO_L stretching mode is IR inactive.

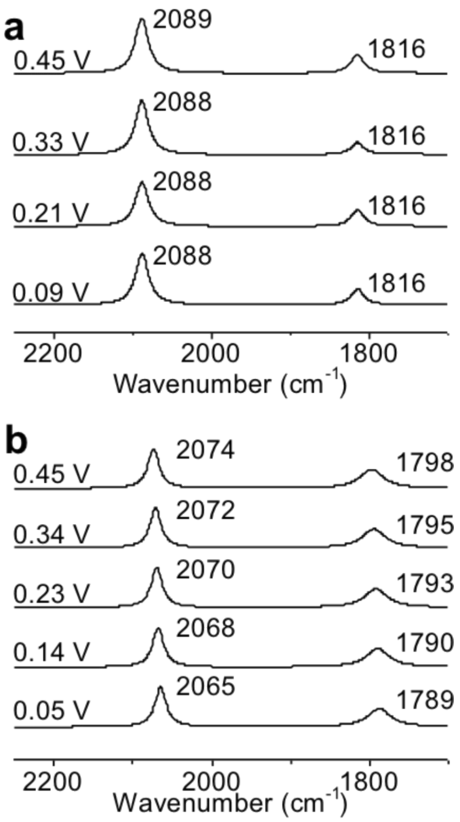

Fig. S3 The calculated IR spectra of (2×2)-3CO α_1 adstructure (a) at 0.09 V — 0.45 V in vacuum and (b) tat 0.05 V — 0.45 V in CO-saturated 0.1 M $HClO_4$ aqueous solution.

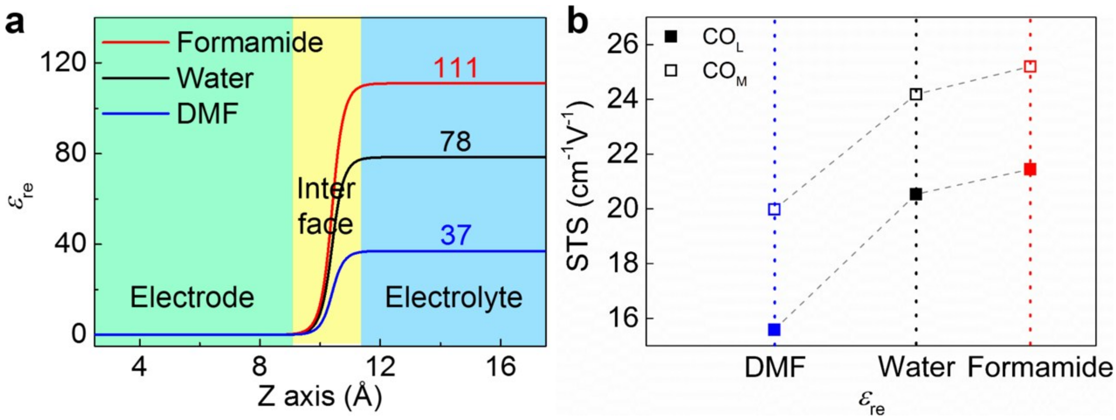

Fig. S4 (a) Distributions of the relative permittivity ε_{re} across the Pt(111)/solution interface with various solvents, dimethyl formamide (DMF), water and formamide solvents along the Z axis. The relative permittivity ε_{re} of the bulk DMF, water, and formamide are 111, 78, and 37, respectively. (b) Dependence of STSs of CO_L and CO_M bands on solvents.

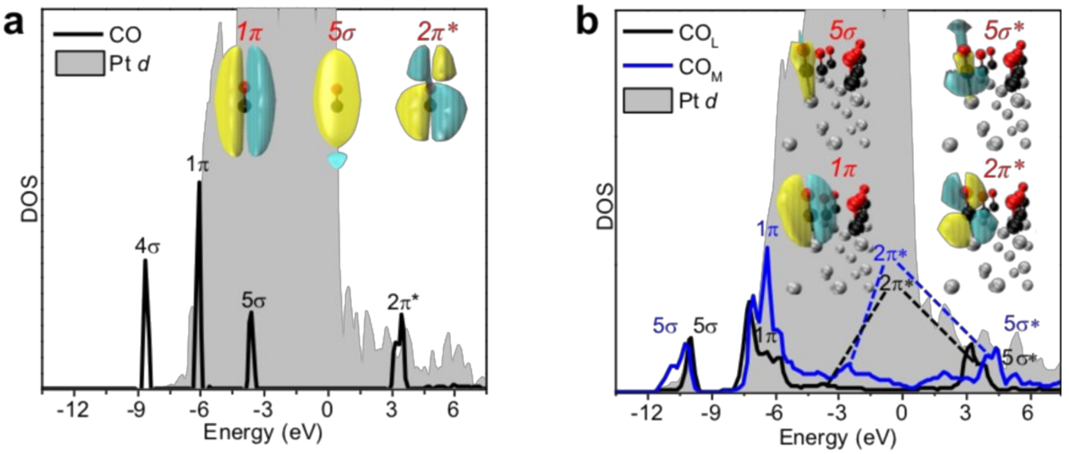

Fig. S5 pDOSs of (a) the isolated CO molecule orbital (black line) with the clean Pt(111) surface *d* orbital (gray filled curve) and (b) the CO_L (black line) and CO_M (blue line) molecule orbitals with Pt(111) surface *d* orbital of Pt(111)(2×2)-3CO α_1 adstructure at 0.23 V vs. SHE. The Fermi level is located at 0 eV. The yellow and cyan parts donate the positive and negative phase of NBO wavefunctions.

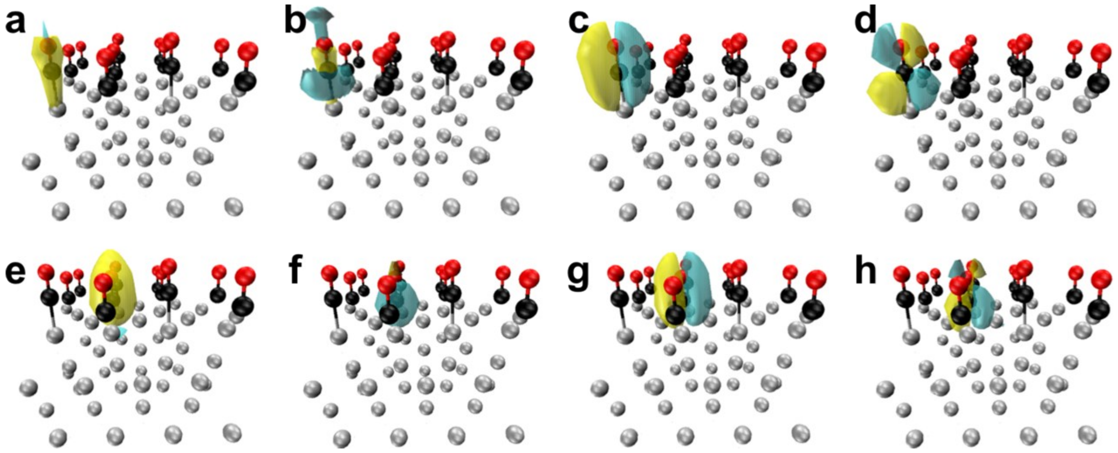

Fig. S6 NBO wavefunctions of the CO_L and CO_M bonds: (a) and (e) denote the 5 σ orbitals; (b) and (f) denote the 5 σ* orbitals; (c) and (g) denote the 1 π orbital; (d) and (h) denote the 2 π* orbitals. The yellow and cyan parts denote the positive and negative phase of the wavefunctions, respectively.

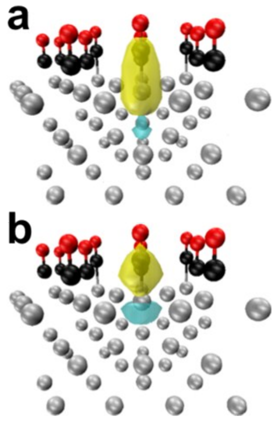

Fig. S7 NBO wavefunctions of the PtC_L bond: (a) and (b) denote the σ bonding orbital and the σ^* anti-bonding orbital, respectively. The yellow and cyan parts denote the positive and negative phase of the wavefunctions, respectively.

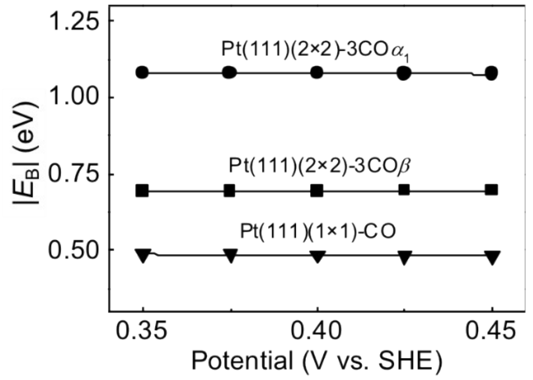

Fig. S8 Absolute values of potential-dependent binding energies $|E_B|$ for the (2×2)-3CO α_1, (2×2)-3CO β and (1×1)-CO adstructures.

Table S1. Slope of the NBO occupancy vs. potential for (2×2)-3CO α_1 adstructure.

	Slope (e/V)
CO_L 5 σ	0.000
CO_L 5 σ^*	-0.001
CO_M 5 σ	0.000
CO_M 5 σ^*	-0.001
CO_L 1 π	0.000
CO_L 2 π^*	-0.019
CO_M 1 π	-0.001
CO_M 2 π^*	-0.024
PtC_L σ	-0.003
PtC_L σ^*	0.000

Table S2. Vibrational frequencies of the (2×2)-3CO α_1 adstructure at 0.45 V *vs*. SHE in a CO-saturated 0.1 M $HClO_4$ aqueous solution under different force convergence criteria (ε_{force}) from 0.08 to 0.005 eVÅ^{-1}.

ε_{force} (eV Å^{-1})	CO_L (cm^{-1})	CO_M (cm^{-1})	PtC_L (cm^{-1})	PtC_M (cm^{-1})
Expt.	2074	1798	473	392
0.08	2070	1797	467	391
0.04	2073	1798	472	392
0.02	2074	1798	473	392
0.01	2074	1798	473	392
0.005	2074	1798	473	392

References

1. G. Kresse and J. Furthmuller, *Phys. Rev. B*, 1996, **54**, 11169-11186.
2. B. Hammer, L. B. Hansen and J. K. Nørskov, *Phys. Rev. B*, 1999, **59**, 7413-7421.

3. G. Kresse and D. Joubert, *Phys. Rev. B*, 1999, **59**, 1758-1775.
4. K. Mathew, R. Sundararaman, K. Letchworth-Weaver, T. Arias and R. G. Hennig, *J. Chem. Phys.*, 2014, **140**, 084106.
5. K. Mathew and R. G. Hennig, *arXiv preprint arXiv:1601.03346*, 2016.
6. J. S. Filhol and M. Neurock, *Angew. Chem. Int. Ed.*, 2006, **45**, 402-406.
7. C. Taylor, R. G. Kelly and M. Neurock, *J. Electrochem. Soc.*, 2006, **153**.
8. *GoldBook*, Standard Hydrogen electrode, http://goldbook.iupac.org/S05917.html.
9. S. N. Steinmann, C. Michel, R. Schwiedernoch and P. Sautet, *Phys. Chem. Chem. Phys.*, 2015, **17**, 13949-13963.
10. J.-L. Fattebert and F. Gygi, *Int. J. Quantum Chem.*, 2003, **93**, 139-147.
11. D. Porezag and M. R. Pederson, *Phys Rev B Condens Matter*, 1996, **54**, 7830-7836.

Appendix I. Calculated geometries

Geometry of Pt(111)(2×2)-3CO α_1

Unit cell			
a (Å)	*c* (Å)	*γ* (°)	
5.61	108.75	120	

Positions			
x (Å)	*y* (Å)	*z* (Å)	Elements
0.00	0.00	0.00	Pt
-1.40	2.43	0.00	Pt
1.40	2.43	0.00	Pt
2.81	0.00	0.00	Pt
2.81	1.62	2.29	Pt
1.40	4.05	2.29	Pt
-1.40	4.05	2.29	Pt
0.00	1.62	2.29	Pt
0.00	3.24	4.58	Pt
1.40	0.81	4.58	Pt
4.21	0.81	4.58	Pt
2.81	3.24	4.58	Pt
0.00	0.00	6.88	Pt
-1.40	2.43	6.88	Pt
1.40	2.43	6.88	Pt
2.81	0.00	6.88	Pt
0.00	0.00	101.88	Pt
-1.40	2.43	101.88	Pt
1.40	2.43	101.88	Pt
2.81	0.00	101.88	Pt
2.81	1.62	104.17	Pt
1.40	4.05	104.17	Pt
-1.40	4.05	104.17	Pt
0.00	1.62	104.17	Pt
0.00	3.24	106.46	Pt
1.40	0.81	106.46	Pt
4.21	0.81	106.46	Pt
2.81	3.24	106.46	Pt
0.00	0.00	8.88	C
0.00	3.24	8.88	C
2.81	1.62	8.88	C
0.00	3.24	99.88	C
2.81	1.62	99.88	C
0.00	0.00	99.88	C
0.00	0.00	10.38	O
0.00	3.24	10.38	O
2.81	1.62	10.38	O
0.00	0.00	98.38	O
0.00	3.24	98.38	O
2.81	1.62	98.38	O

Geometry of Pt(111)(2×2)-3CO α_2

Positions			
x (Å)	*y* (Å)	*z* (Å)	Elements
2.11	3.65	0.00	Pt
3.51	1.22	0.00	Pt
0.70	1.22	0.00	Pt
-0.70	3.65	0.00	Pt
2.11	0.41	2.29	Pt
0.70	2.84	2.29	Pt
3.51	2.84	2.29	Pt
4.91	0.41	2.29	Pt
-0.70	2.03	4.58	Pt
-2.11	4.46	4.58	Pt
0.70	4.46	4.58	Pt
2.11	2.03	4.58	Pt
2.11	3.65	6.88	Pt
3.51	1.22	6.88	Pt
0.70	1.22	6.88	Pt
-0.70	3.65	6.88	Pt
2.11	3.65	101.87	Pt
3.51	1.22	101.87	Pt
0.70	1.22	101.87	Pt
-0.70	3.65	101.87	Pt
2.11	0.41	104.17	Pt
0.70	2.84	104.17	Pt
3.51	2.84	104.17	Pt
4.91	0.41	104.17	Pt
-0.70	2.03	106.46	Pt
-2.11	4.46	106.46	Pt
0.70	4.46	106.46	Pt
2.11	2.03	106.46	Pt
0.00	0.00	8.88	C
3.16	1.42	8.88	C
-0.35	3.44	8.88	C
0.00	0.00	99.87	C
3.16	1.42	99.87	C
-0.35	3.44	99.87	C
0.00	0.00	10.38	O
3.16	1.42	10.38	O
-0.35	3.44	10.38	O
0.00	0.00	98.37	O
3.16	1.42	98.37	O
-0.35	3.44	98.37	O

Geometry of Pt(111)(2×2)-3CO *β*

Positions			
x (Å)	*y* (Å)	*z* (Å)	Elements
0.00	0.00	0.00	Pt
-1.40	2.43	0.00	Pt
1.40	2.43	0.00	Pt
2.81	0.00	0.00	Pt
2.81	1.62	2.29	Pt
1.40	4.05	2.29	Pt
-1.40	4.05	2.29	Pt
0.00	1.62	2.29	Pt
0.00	3.24	4.58	Pt
1.40	0.81	4.58	Pt
4.21	0.81	4.58	Pt
2.81	3.24	4.58	Pt
0.00	0.00	6.88	Pt
-1.40	2.43	6.88	Pt
1.40	2.43	6.88	Pt
2.81	0.00	6.88	Pt
0.00	0.00	101.88	Pt
-1.40	2.43	101.88	Pt
1.40	2.43	101.88	Pt
2.81	0.00	101.88	Pt
2.81	1.62	104.17	Pt
1.40	4.05	104.17	Pt
-1.40	4.05	104.17	Pt
0.00	1.62	104.17	Pt
0.00	3.24	106.46	Pt
1.40	0.81	106.46	Pt
4.21	0.81	106.46	Pt
2.81	3.24	106.46	Pt
0.00	0.00	8.88	C
-1.40	2.43	8.88	C
1.40	2.43	8.88	C
0.00	0.00	99.88	C
-1.40	2.43	99.88	C
1.40	2.43	99.88	C
-1.40	2.43	10.38	O
0.00	0.00	10.38	O
1.40	2.43	10.38	O
0.00	0.00	98.38	O
-1.40	2.43	98.38	O
1.40	2.43	98.38	O

Geometry of Pt(111)(1×1)-CO

Positions			
x (Å)	*y* (Å)	*z* (Å)	Elements
2.11	3.65	0.00	Pt
3.51	1.22	0.00	Pt
0.70	1.22	0.00	Pt
-0.70	3.65	0.00	Pt
2.11	0.41	2.29	Pt
0.70	2.84	2.29	Pt
3.51	2.84	2.29	Pt
4.91	0.41	2.29	Pt
-0.70	2.03	4.58	Pt
-2.11	4.46	4.58	Pt
0.70	4.46	4.58	Pt
2.11	2.03	4.58	Pt
2.11	3.65	6.88	Pt
3.51	1.22	6.88	Pt
0.70	1.22	6.88	Pt
-0.70	3.65	6.88	Pt
2.11	3.65	101.88	Pt
3.51	1.22	101.88	Pt
0.70	1.22	101.88	Pt
-0.70	3.65	101.88	Pt
2.11	0.41	104.17	Pt
0.70	2.84	104.17	Pt
3.51	2.84	104.17	Pt
4.91	0.41	104.17	Pt
-0.70	2.03	106.46	Pt
-2.11	4.46	106.46	Pt
0.70	4.46	106.46	Pt
2.11	2.03	106.46	Pt
2.11	3.65	8.88	C
3.51	1.22	8.88	C
0.70	1.22	8.88	C
-0.70	3.65	8.88	C
2.11	3.65	99.88	C
3.51	1.22	99.88	C
0.70	1.22	99.88	C
-0.70	3.65	99.88	C
2.11	3.65	10.38	O
3.51	1.22	10.38	O
0.70	1.22	10.38	O
-0.70	3.65	10.38	O
2.11	3.65	98.38	O
3.51	1.22	98.38	O
0.70	1.22	98.38	O
-0.70	3.65	98.38	O

Geometry of Pt(111)(√19×√19)-13CO α_1

Unit cell		
a (Å)	*c* (Å)	*γ* (°)
12.24	108.75	120

Positions

x (Å)	*y* (Å)	*z* (Å)	Elements	*x* (Å)	*y* (Å)	*z* (Å)	Elements
0.00	0.00	0.00	Pt	6.44	6.32	4.58	Pt
5.15	2.23	0.00	Pt	1.93	9.67	4.58	Pt
0.64	5.58	0.00	Pt	0.00	0.00	6.88	Pt
-3.86	8.92	0.00	Pt	5.15	2.23	6.88	Pt
5.80	7.81	0.00	Pt	0.64	5.58	6.88	Pt
7.41	0.56	0.00	Pt	-3.86	8.92	6.88	Pt
2.90	3.90	0.00	Pt	5.80	7.81	6.88	Pt
-1.61	7.25	0.00	Pt	7.41	0.56	6.88	Pt
8.05	6.14	0.00	Pt	2.90	3.90	6.88	Pt
3.54	9.48	0.00	Pt	-1.61	7.25	6.88	Pt
0.32	2.79	0.00	Pt	8.05	6.14	6.88	Pt
9.98	1.67	0.00	Pt	3.54	9.48	6.88	Pt
5.47	5.02	0.00	Pt	0.32	2.79	6.88	Pt
0.97	8.37	0.00	Pt	9.98	1.67	6.88	Pt
2.58	1.12	0.00	Pt	5.47	5.02	6.88	Pt
-1.93	4.46	0.00	Pt	0.97	8.37	6.88	Pt
7.73	3.35	0.00	Pt	2.58	1.12	6.88	Pt
3.22	6.69	0.00	Pt	-1.93	4.46	6.88	Pt
-1.29	10.04	0.00	Pt	7.73	3.35	6.88	Pt
1.93	2.60	2.29	Pt	3.22	6.69	6.88	Pt
-2.58	5.95	2.29	Pt	-1.29	10.04	6.88	Pt
7.08	4.83	2.29	Pt	0.00	0.00	101.88	Pt
2.58	8.18	2.29	Pt	5.15	2.23	101.88	Pt
4.19	0.93	2.29	Pt	0.64	5.58	101.88	Pt
-0.32	4.28	2.29	Pt	-3.86	8.92	101.88	Pt
9.34	3.16	2.29	Pt	5.80	7.81	101.88	Pt
4.83	6.51	2.29	Pt	7.41	0.56	101.88	Pt
0.32	9.85	2.29	Pt	2.90	3.90	101.88	Pt
6.76	2.05	2.29	Pt	-1.61	7.25	101.88	Pt
2.25	5.39	2.29	Pt	8.05	6.14	101.88	Pt
-2.25	8.74	2.29	Pt	3.54	9.48	101.88	Pt
7.41	7.62	2.29	Pt	0.32	2.79	101.88	Pt
-0.64	1.49	2.29	Pt	9.98	1.67	101.88	Pt
9.02	0.37	2.29	Pt	5.47	5.02	101.88	Pt
4.51	3.72	2.29	Pt	0.97	8.37	101.88	Pt
0.00	7.06	2.29	Pt	2.58	1.12	101.88	Pt
-4.51	10.41	2.29	Pt	-1.93	4.46	101.88	Pt
5.15	9.30	2.29	Pt	7.73	3.35	101.88	Pt
-1.29	2.97	4.58	Pt	3.22	6.69	101.88	Pt
8.37	1.86	4.58	Pt	-1.29	10.04	101.88	Pt
3.86	5.21	4.58	Pt	1.93	2.60	104.17	Pt
-0.64	8.55	4.58	Pt	-2.58	5.95	104.17	Pt
0.97	1.30	4.58	Pt	7.08	4.83	104.17	Pt

10.63	0.19	4.58	Pt	2.58	8.18	104.17	Pt
6.12	3.53	4.58	Pt	4.19	0.93	104.17	Pt
1.61	6.88	4.58	Pt	-0.32	4.28	104.17	Pt
-2.90	10.23	4.58	Pt	9.34	3.16	104.17	Pt
6.76	9.11	4.58	Pt	4.83	6.51	104.17	Pt
3.54	2.42	4.58	Pt	0.32	9.85	104.17	Pt
-0.97	5.76	4.58	Pt	6.76	2.05	104.17	Pt
8.69	4.65	4.58	Pt	2.25	5.39	104.17	Pt
4.19	7.99	4.58	Pt	-2.25	8.74	104.17	Pt
5.80	0.74	4.58	Pt	7.41	7.62	104.17	Pt
1.29	4.09	4.58	Pt	-0.64	1.49	104.17	Pt
-3.22	7.44	4.58	Pt	9.02	0.37	104.17	Pt
4.51	3.72	104.17	Pt	9.91	2.42	99.88	C
0.00	7.06	104.17	Pt	-1.47	5.76	99.88	C
-4.51	10.41	104.17	Pt	7.59	4.83	99.88	C
5.15	9.30	104.17	Pt	0.85	3.35	99.88	C
-1.29	2.97	106.46	Pt	5.26	7.25	99.88	C
8.37	1.86	106.46	Pt	3.18	0.93	99.88	C
3.86	5.21	106.46	Pt	2.94	9.66	99.88	C
-0.64	8.55	106.46	Pt	-0.43	8.92	99.88	C
0.97	1.30	106.46	Pt	1.90	6.51	99.88	C
10.63	0.19	106.46	Pt	4.22	4.09	99.88	C
6.12	3.53	106.46	Pt	6.54	1.67	99.88	C
1.61	6.88	106.46	Pt	0.00	0.00	10.38	O
-2.90	10.23	106.46	Pt	-3.79	8.18	10.38	O
6.76	9.11	106.46	Pt	9.91	2.42	10.38	O
3.54	2.42	106.46	Pt	-1.47	5.76	10.38	O
-0.97	5.76	106.46	Pt	7.59	4.83	10.38	O
5.80	0.74	106.46	Pt	0.85	3.35	10.38	O
1.29	4.09	106.46	Pt	5.26	7.25	10.38	O
8.69	4.65	106.46	Pt	3.18	0.93	10.38	O
4.19	7.99	106.46	Pt	2.94	9.66	10.38	O
-3.22	7.44	106.46	Pt	-0.43	8.92	10.38	O
6.44	6.32	106.46	Pt	1.90	6.51	10.38	O
1.93	9.67	106.46	Pt	4.22	4.09	10.38	O
0.00	0.00	8.88	C	6.54	1.67	10.38	O
-3.79	8.18	8.88	C	0.00	0.00	98.38	O
9.91	2.42	8.88	C	-3.79	8.18	98.38	O
-1.47	5.76	8.88	C	9.91	2.42	98.38	O
7.59	4.83	8.88	C	-1.47	5.76	98.38	O
0.85	3.35	8.88	C	7.59	4.83	98.38	O
5.26	7.25	8.88	C	0.85	3.35	98.38	O
3.18	0.93	8.88	C	5.26	7.25	98.38	O
2.94	9.66	8.88	C	3.18	0.93	98.38	O
-0.43	8.92	8.88	C	2.94	9.66	98.38	O
1.90	6.51	8.88	C	-0.43	8.92	98.38	O
4.22	4.09	8.88	C	1.90	6.51	98.38	O
6.54	1.67	8.88	C	4.22	4.09	98.38	O

0.00	0.00	99.88	C	6.54	1.67	98.38	O
-3.79	8.18	99.88	C				
